# A New Electrochemical Sensor for Dopamine Detection Based on Reduced Graphene Oxide Modified with Samarium Oxide Nanoparticles

**DOI:** 10.1021/acsomega.5c08166

**Published:** 2025-11-14

**Authors:** Rodrigo Vieira Blasques, Vinicius Aparecido Pedro Oliani da Silva, Amanda Caroline Nascimento Sousa, Tatiana Maria Barreto de Freitas, Leliz Ticona Arenas, Glauber Cruz, Marcelo Barcellos da Rosa, Gabriel Braga Marques Teobaldo, Matheus Henrique Martins, Fabio Luiz Pissetti, Rita de Cássia Mendonça de Miranda, Luís Cláudio Nascimento da Silva, Paulo César Mendes Villis

**Affiliations:** † Electrochemistry and Biotechnology Laboratory, 125287 University of CEUMA - UNICEUMA, 65065-470 São Luís, MA, Brazil; ‡ Laboratory of Sensors, Nanomedicine and Nanostructured Materials, 67828 Federal University of São Carlos, 13600-970 Araras, Brazil; § Laboratory of Solids and Surfaces, Institute of Chemistry, 28124 Federal University of Rio Grande do Sul − UFRGS, 91501-970 Porto Alegre, RS, Brazil; ∥ Processes and Thermochemical Systems Laboratory (LPSisTer), Department of Mechanical Engineering, 37892 Federal University of Maranhão (UFMA), 65085-580 São Luís, Maranhão, Brazil; ⊥ Department of Chemistry, 28118 Federal University of Santa Maria - UFSM, Av. Roraima, 1000, 97105-900 Santa Maria, RS, Brazil; # Institute of Chemistry, 74347 Federal University of Alfenas, Rua Gabriel Monteiro da Silva, 700, 37130-001 Alfenas, MG, Brazil; ∇ Institute of Physics, 28133 University of São Paulo, 05508-090 São Paulo, Brazil

## Abstract

This work reports the fabrication of a novel electrochemical sensor leveraging reduced graphene oxide (rGO) modified with samarium oxide (Sm_2_O_3_) nanoparticles to enhance dopamine (DA) detection. The primary goal was to create a sensitive and selective platform capable of distinguishing DA in complex biological environments. The sensor was synthesized using a hydrothermal method to form rGO/Sm_2_O_3_ composites, followed by characterization employing scanning electron microscopy (SEM), X-ray diffraction (XRD), transmission electron microscopy (TEM), and high-resolution TEM (HR-TEM) and Raman spectroscopy to confirm morphological and structural integrity. Electrochemical assessments were conducted via cyclic voltammetry and square wave voltammetry, with the latter exhibiting an optimal response characterized by a linear range from 0.5 to 20.0 μmol L^–1^ and a limit of detection (LOD) of 0.030 μmol L^–1^. Comparative analyses highlighted the sensor’s enhanced performance over conventional materials, with a 10-fold improvement in electron transfer rate, resulting from the higher electroactive area and the inherent functional properties of Sm_2_O_3_. The high selectivity was confirmed by testing against common interfering substances, and the sensor demonstrated reliable DA detection in synthetic human serum, achieving recovery rates between 95.27% and 99.39%. The findings suggested that the rGO/Sm_2_O_3_ sensor offers a robust, low-cost, and effective solution for DA detection with potential applications in clinical diagnostics and real-time monitoring of neurotransmitter levels in complex biological systems. This advancement underscores the relevance of integrating rare-earth oxides in sensors technology to address selectivity and sensitivity challenges in neurochemical analysis.

## Graphical abstract

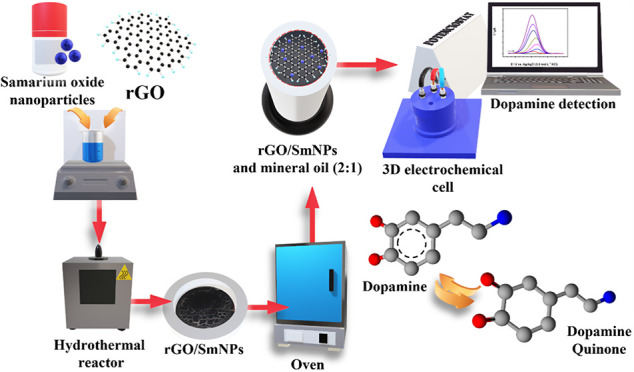

## 1 Introduction

Selective and sensitive detection of dopamine (DA) is essential in neuroscience studies, owing to its pivotal role in neurotransmission within the central nervous system and its association with diseases such as Parkinson’s and depression.^1–3^ DA is closely related to several neurological functions, including mood regulation, motivational behavior, and learning.^4^ Furthermore, the environment can drastically influence DA levels, directly affecting the neural system and leading to potential neuropsychiatric and behavioral disorders.^5^ Exposure to air pollutants, for example, is associated with neurobehavioral changes, which can negatively impact mental health. As Weitekamp and Hofmann^6^ discussed, air pollution can compromise neural function and is linked to increased cases of depression and autism spectrum disorders. This environmental factor makes the accurate detection of neurotransmitters such as DA even more relevant, since it allows to evaluate how the external environment can influence fundamental brain functions.^7^

Due to this importance, accurate analysis of DA in the central nervous system can offer deep insights into the identification and management of neurological diseases. However, measuring DA represents a constant challenge in neuroscience and biomedicine as a result of its limited concentration in biofluids and the interference caused by structurally similar species. Several conventional techniques have been widely used for this purpose, such as microdialysis, chemical dye-based methods, and downstream signal-based methods.^8^ Despite their high accuracy and specificity, these methodologies often require sophisticated instrumentation, highly skilled operators, and complex laboratory procedures, including sample extraction and purification steps. On the other hand, the interest in more accessible and efficient alternative methods, such as electrochemistry, stands out for offering operational simplicity, low cost, miniaturization potential, and excellent analytical performance, while maintaining high levels of selectivity and sensitivity.^9^

Advances in electrochemical sensors modified with nanomaterials,^10,11^ including reduced graphene oxide (rGO) and rare-earth metal oxides (e.g., Sm_2_O_3_), provide significant improvements in the selectivity and sensitivity of DA sensors, enhancing their ability to distinguish DA from other biological interferents.^12^ Rare-earth ions have attracted considerable attention as dopants owing to their unique spectroscopic behavior and versatile applications in optoelectronic technologies such as fiber amplifiers, laser systems, upconversion devices, and luminescent phosphors.^13^

Because of their superior sensitivity, selectivity, and fast response characteristics, electrochemical sensors have become promising tools for applications in environmental analysis and clinical detection. However, they still face significant challenges in effectively selecting compounds in complex matrices, where interference can compromise accuracy. These challenges are often associated with the need to optimize electrode materials and designs to improve sensor performance, especially in media containing various chemical interferents.^14^

In this perspective, the modification of electrodes with nanomaterials such as rGO, which is valued for its high surface area and excellent electrical conductivity, has proven to be an effective strategy to improve the efficiency of DA sensors, allowing a higher level of selectivity.^15,16^ Furthermore, the combination of rGO with rare-earth oxides significantly improves the electrocatalytic activity of these sensors. For these reasons, Sm_2_O_3_, integrated with rGO, presents itself as a promising modifier for the electrochemical detection of DA in complex biological environments. From this combination, the sensors gain not only in sensitivity and selectivity, but also in long-term stability, an essential factor for clinical applications and real-time analysis.^17^

The literature addresses several works for DA detection using graphene and modifications with materials such as carbon nanotubes,^18^ poly­(3,4-ethylenedioxythiophene)/poly­(styrene-4-sulfonate) (PEDOT/PSS),^18^ platinum–silver,^19^ tin dioxide and gold nanoparticles.^20^ The innovative combination of rGO and Sm_2_O_3_ represents a promising and effective approach for advancing DA electrochemical sensors, enabling breakthroughs in neuroscience applications that require high accuracy and reliability under challenging sensing conditions. Other materials are also used in DA detection, such as macromolecule–nanoparticle-based hybrid materials^21^ and phthalocyanines.^22^

The combination of Sm_2_O_3_ with rGO represents an innovative strategy for the development of electrochemical sensors for DA detection. This synergistic approach leverages the high electrical conductivity of rGO and the excellent catalytic activity, chemical stability, and high dielectric constant of Sm_2_O_3_, a rare-earth oxide widely applied in optoelectronic and sensing devices. While Sm_2_O_3_ has been extensively studied in gas sensing and resistive memory applications, its use in the electrochemical detection of neurotransmitters in complex biological media remains underexplored. The integration of these materials results in a hybrid sensing platform with enhanced sensitivity, selectivity, and long-term stability, holding strong potential for clinical diagnostics and neuroscience research.

## 2 Experimental Section

### 2.1 Reagents and Solutions

All chemicals used in this study were of analytical grade and used without any additional purification. High-purity samarium­(III) oxide nanopowder (Sm_2_O_3_ ≤ 100 nm, 99%, Sigma-Aldrich) and graphene powder (99% w/w) were employed as key materials. Potassium ferricyanide K_3_[Fe­(CN)_6_] and potassium ferrocyanide K_4_[Fe­(CN)_6_] (99% w/w) were also supplied by Sigma-Aldrich and used as standard redox mediators in the electrochemical analyses. Ethanol (99.5% v/v) and dopamine hydrochloride (99% w/w) were obtained from Sigma-Aldrich. Ultrapure water (Milli-Q), with resistivity >18 MΩ cm, was used to prepare all the aqueous solutions. 0.1 mol L^–1^ phosphate buffer saline solution (PBS) (pH 7.0) was used as the supporting electrolyte and prepared from a mixture mono- and dibasic sodium phosphate anhydrous (99% w/w); potassium chloride (99% w/w) was obtained from Sigma-Aldrich. Human serum was obtained from Sigma-Aldrich. DA stock solution (1 mmol L^–1^) was freshly prepared by dissolving in PBS prior to experiments. For the construction of the calibration curve of DA, the stock solution was diluted in different concentrations in PBS again (0.5, 1.0, 3.0, 5.0, 10.0, 15.0, and 20.0 μmol L^–1^). For DA analysis, human serum was diluted 1:100 in PBS at pH 7.0. Then, the samples were spiked with three different DA concentrations (0.5, 1.0, and 3.0 μmol L^–1^). For the electrochemical characterization, 5.0 mmol of L^–1^ [Fe­(CN)_6_]^3–/4–^ in 0.1 mol L^–1^ KCl was used as the electrochemical probe. The portable electrochemical cell was fabricated using an acrylonitrile butadiene styrene (ABS) filament with nonconductive properties, supplied by 3DLAB enterprise (Minas Gerais, Brazil).

### 2.2 Synthesis and Preparation of the rGO/SmNPS Electrode

The rGO/SmNPs material was synthesized through a controllable hydrothermal method (Figure 1). Initially, 0.93 mol L^–1^ of dispersed samarium oxide nanoparticles were added to 40 mL of ultrapure water and stirred for 30 min, followed by the addition of 1 mol L^–1^ KOH liquid solution dropwise until the pH was adjusted to 10 (Figure 1, step A). Then, 2 g of rGO was dissolved in 20 mL of ethanol and added to the previous solution under magnetic stirring for 30 min to form a homogeneous solution (Figure 1, step B). After this procedure, the mixed solution was transferred to a hydrothermal reactor with a capacity of 100 mL, and maintained at 170 °C for 3 h (Figure 1, step C). Finally, the synthesized material was washed with ethanol and DI water and dried in an oven at 70 °C for 6 h (Figure 1, step D). After drying, the material was ground and the working electrode was prepared from a paste using a 2:1 mixture of rGO/SmNPs and mineral oil (Figure 1, step E). After the electrode was prepared, it was applied for DA detection (Figure 1, step F).

**Figure 1:**
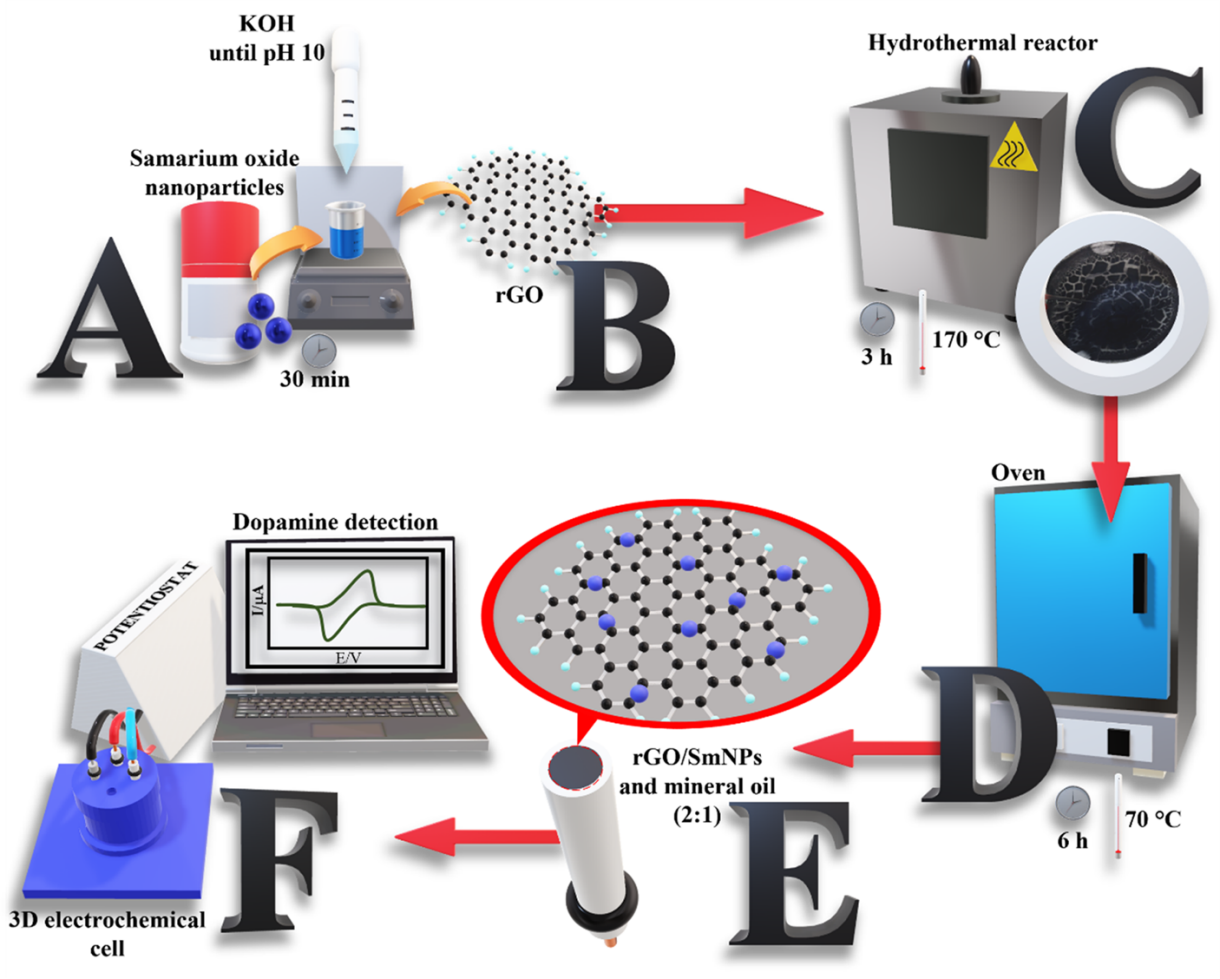
Schematic representation for synthesis and application of rGO/SmNPs.

### 2.3 Instrumental and Apparatus

Transmission electron microscopy (TEM) and high-resolution TEM (HR-TEM) images were acquired using a JEOL JEM 2100 microscope (Peabody) operating at 200 kV. The samples were prepared by drop-casting 5 μL of the material in 2-propanol onto copper grids. For high-angle annular dark-field scanning transmission electron microscopy (HAADF-STEM) imaging, elemental mapping was performed by using Aztec software. ImageJ software was used for the calculation of the interplanar distance. Textural characterization was performed using N_2_ adsorption–desorption isotherms at liquid N_2_ boiling point temperature, using a Tristar Kr 3020 Micromeritics equipment. The samples were previously degassed at 120 °C under vacuum for 12 h. The specific surface area (*S*_BET_) was determined by the BET method (Brunauer, Emmett, and Teller), which is a multipoint technique.^23^ The pore size and volume distribution were obtained using DFT (density functional theory).^23^ The Raman spectra were obtained using a Bruker Senterra confocal Raman microscope (Ettlingen, Germany) fitted with a thermoelectrically cooled CCD camera (Bruker/Andor, 1024 × 256 pixels) and coupled to an Olympus BX-51 microscope. The samples were spread on a glass microscope slide, and the spectra were produced using a 532 nm laser line (diode laser), which were focused on the samples by a 50× Olympus objective lens (NA 0.75). The spectra were produced at 4 cm^–1^ resolution. Laser power and accumulations were tuned according to the sample for a better signal/noise ratio. Visual inspections of the samples and comparisons of spectra obtained with different laser powers were performed to ensure that the samples were not affected by the laser intensity. The best results for this technique were obtained using a 2 mW laser power and 15 s integration time. Spectra of pure rGO samples were obtained using three coadditions, and rGO/SmNPs samples required six coadditions.

The electrochemical cell used was three-dimensional (3D) printed using a Sethi3D S3 printer (Campinas, Brazil), controlled by Simplify 3D software, employing the fused deposition modeling (FDM) technique to manufacture the structures. The use of 3D printing techniques enables the prototyping of devices that meet specific needs and purposes for a moment of application. In this work, the possibility of a robust, stable, and easy-to-handle device for in situ application is presented (Figure S1).

### 2.4 Electrochemical Investigation

The working electrode was fabricated by mixing 50 mg of rGO or rGO/SmNPs with a small amount of mineral oil (approximately 2.0 × 10^–2^ cm^–3^) to form a homogeneous paste. The resulting mixture was carefully packed into the cavity of a poly­(tetrafluoroethylene) (PTFE) tube to obtain a smooth and uniform surface. Electrochemical characterization was carried out using a PGSTAT101 potentiostat/galvanostat (Metrohm Autolab, Eco Chemie) operated through NOVA software (version 2.1.4). A classic three-electrode cell (Figure S1) was used, consisting of a carbon paste electrode as the working electrode (6 mm in diameter), a platinum wire as the auxiliary electrode, and a silver/silver chloride electrode (Ag/AgCl, KCl 3.0 mol L^–1^) as the reference electrode. Phosphate-buffered saline (PBS, 0.1 mol L^–1^, pH 7.0) served as the supporting electrolyte in all electrochemical measurements. All measurements were performed in triplicate, and means and standard deviation (RSD) were calculated.

## 3 Results and Discussion

### 3.1 Characterization of the Proposed Material

The morphology and topology of rGO/SmNPs were studied by TEM (HR-TEM), and the results obtained are shown in Figure 2. The micrograph in Figure 2A of rGO/SmNPs reveals an irregular structure, a characteristic feature of graphene sheets functionalized^24^ with dispersed Sm_2_O_3_ nanoparticles. This irregularity can be attributed to structural defects induced by chemical functionalization and interaction with Sm_2_O_3_ nanoparticles. Elemental mapping (Figure 2B–D) confirms the homogeneous distribution of the elements carbon (C), oxygen (O), and samarium (Sm), evidencing an efficient interaction between the components.

**Figure 2:**
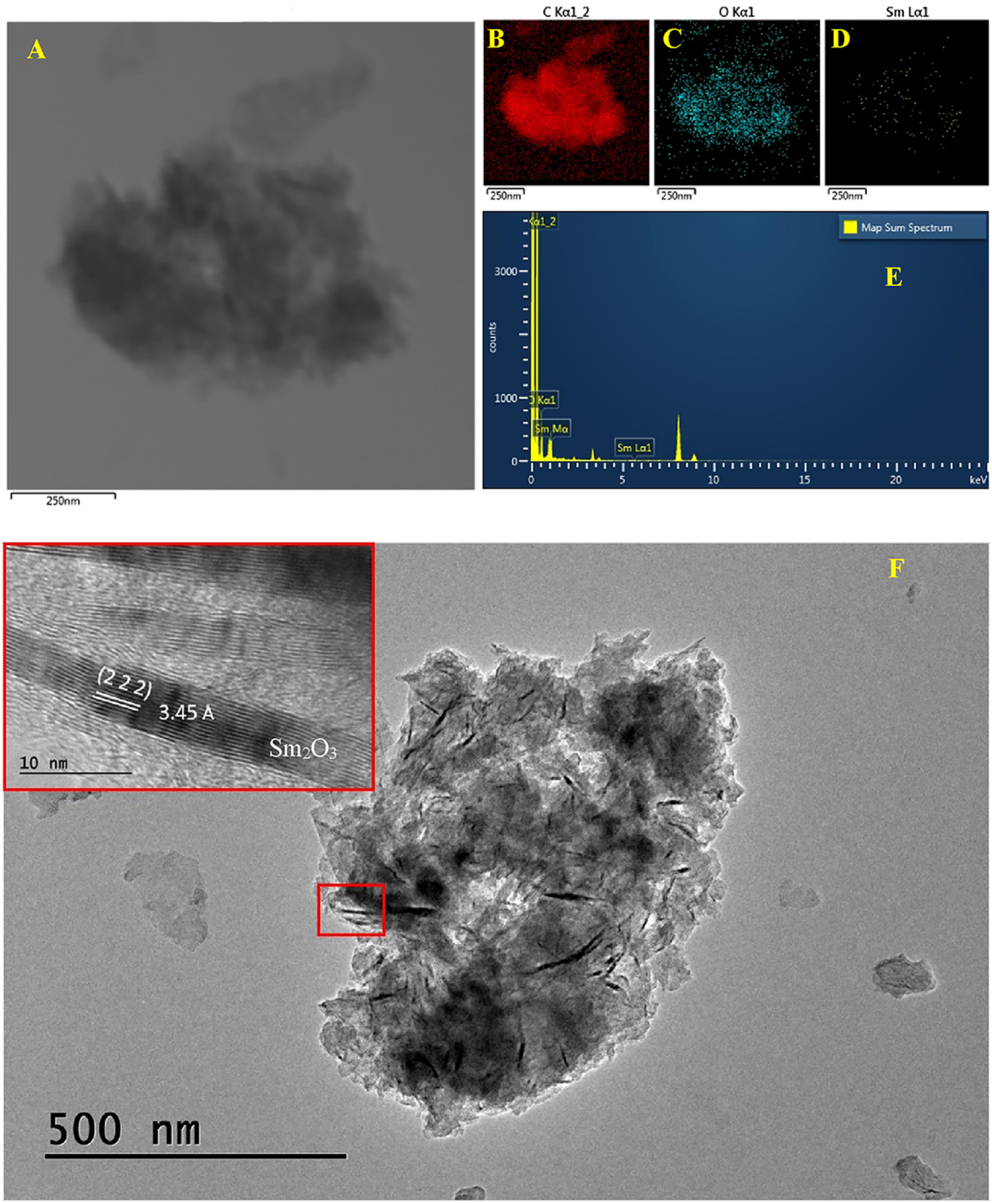
(A) HR-TEM image of the rGO/SmNPs. Elemental mapping (B) carbon (red), (C) oxygen (cyan), and (D) samarium (yellow). (E) EDS spectra and elemental composition for rGO/SmNPs. (F) Lattice fringes of rGO/SmNPs.

The presence of samarium (Sm) was confirmed both by EDS elemental mapping (Figure 2D), where its homogeneous distribution over the surface is observed, and by spectral analysis (Figure E), which highlights characteristic Sm peaks (Sm Mα and Sm Lα1), indicating its successful incorporation into the rGO matrix. This evidence is reinforced by the Sm Mα1 peak with a notable intensity at 1.5 keV. The EDS spectrum (Figure 2E) confirms the expected chemical composition of the composite: the most intense Sm peak suggests a significant amount of incorporated Sm_2_O_3_, while the carbon and oxygen signals are associated with the graphene structure and its surface functionalization. Although elemental mapping by HR-TEM detected Sm at a lower intensity, this observation can be attributed to the inherent limitations of the technique, such as the reduced interaction volume of the electron beam and the sample preparation, which may not adequately represent the distribution of elements present in low concentration or distributed heterogeneously. In contrast, SEM-EDS analyses, which cover larger areas and have a greater interaction volume, evidenced the presence of Sm more clearly. To complement the presented data and provide a more comprehensive view of the elemental composition, the SEM-EDS results, including spectra and distribution maps of Sm, are included in the Supporting Information (Figure S2).

Figure 2F presents a larger-scale micrograph of the overall structure of the material, confirming the dispersion of the nanoparticles in a graphene matrix. The magnification in the highlighted area (red box) provides a high-resolution analysis of the crystal structure of the Sm_2_O_3_ nanoparticles. The (222) crystallographic plane with a spacing of 3.45 Å is clearly identified, indicating high crystallinity. This high crystallinity is essential because it confers well-defined electronic and optical properties to the nanoparticles, making them suitable for applications in sensors and electronic devices.

Textural characteristics of the rGO and rGO/SmNPs materials were studied using the N_2_ desorption-adsorption isotherms. It is observed in Figure 3A that the curves of the materials do not coincide, forming a hysteresis loop, which is typical of mesoporous materials.^25^ The presence of hysteresis suggests that the material has mesoporous pores (2–50 nm), where capillary condensation occurs.^26^ Furthermore, the difference between the adsorption and desorption curves can be associated with the phenomenon of filling and emptying of pores of different sizes and shapes. Furthermore, in the DFT pore size distribution^23^ illustrated in Figure 3B, it is evident that the black curve (rGO) exhibits a distribution of pores with pronounced peaks around 1 to 2 nm, suggesting the predominance of micropores (pores smaller than 2 nm). The red curve (rGO/SmNPs) also evidences a peak around 1 to 2 nm, but with a broader and more significant distribution throughout the range from 3 to 10 nm, indicating the presence of mesopores (pores between 2 and 50 nm). This may indicate structural modifications in the rGO/SmNPs material, attributed to hydrothermal synthesis, a method known to promote the formation of mesoporous structures.^27,28^ Furthermore, the BET-specific surface area (*S*_BET_) for the rGO material was 660 ± 10 m^2^ g^–1^, while the rGO/SmNPs material showed lower values, 555 ± 10 m^2^ g^–1^. The results obtained demonstrate that a new material was formed through a hydrothermal synthesis.

**Figure 3:**
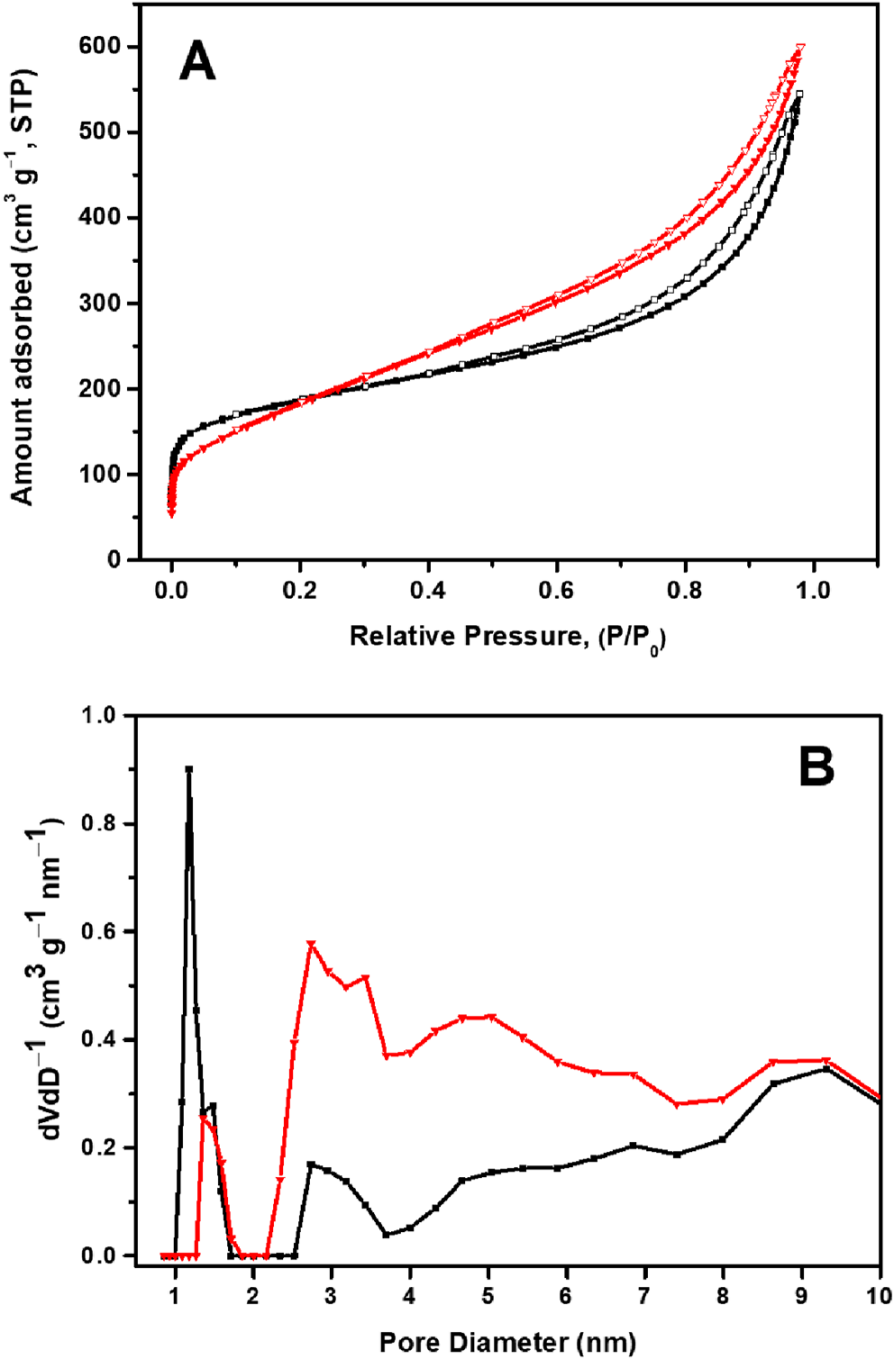
(A) N_2_ adsorption–desorption isotherms and (B) DFT pore size distribution curve for the (**−**) rGO and (**−**) rGO/SmNPs samples.

Figure 4A shows the X-ray diffractogram (XRD) obtained for rGO and rGO/SmNPs. For rGO, a diffraction peak (2θ) located at 26.42° is very sharp. This peak corresponds to the crystalline plane (002), which is indicative of the organization of the stacked graphene sheets^29,30^ and is in accordance with standard diffraction data for reduced graphene oxide (rGO) as indicated in the data sheet JCPDS (Joint Committee on Powder Diffraction Standards) n° 75–2078. Samarium oxide (Sm_2_O_3_) is a rare-earth oxide that has a crystal structure different from that of graphene. Thus, it presents multiple 2θ angles, generally around 28.30°, 32.52°, 41.40°, 57.87°, 60.41°, and 72.82°, which are attributed to the corresponding planes (2 2 2), (4 0 0), (2 0 0), (6 2 2), (2 2 0), and (3 1 1) of the cubic structure of Sm_2_O_3_ with space group *Ia*3̅, respectively.^31–33^ These values are in good agreement with JCPDS standard sheet no. 03–065–3183, confirming the presence of Sm_2_O_3_.

**Figure 4:**
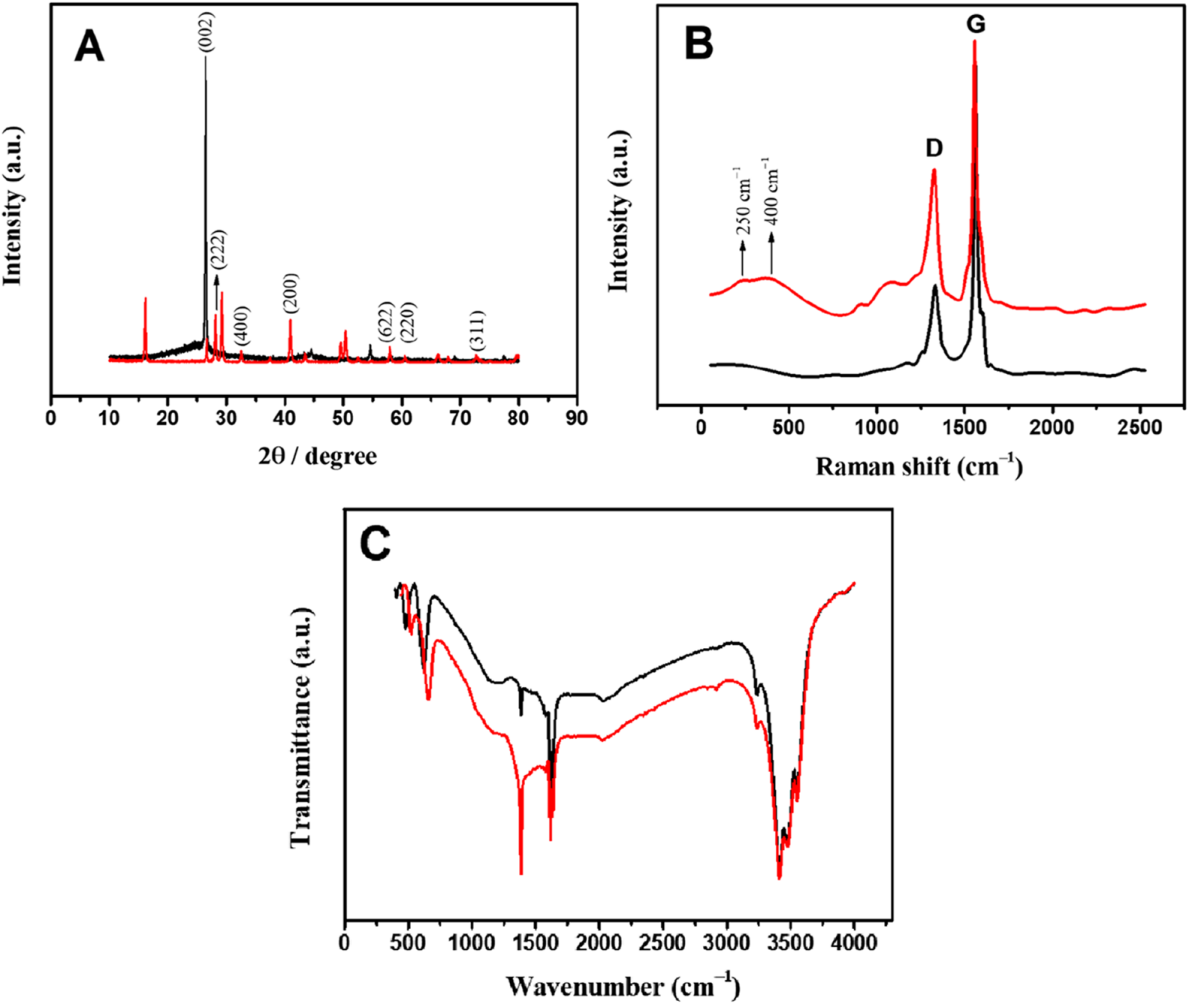
(A) XRD pattern, (B) Raman spectra, and (C) FTIR spectra for (**−**) rGO and (**−**) rGO/SmNPs.

Figure 4B shows the Raman spectra of rGO and the rGO/SmNPs. It is possible to observe for both materials the presence of D and G bands at 1332 and 1561 cm^–1^, respectively. The G band is associated with a defective graphitic structure (sp^2^) and the D band is associated with disorder (sp^3^), which is formed by vibrational forms that become active when there are defects and functionalizations, such as the presence of −OH and −COOH groups, in the hexagonal planes of these structures.^34–36^ The peaks observed at 250 and 400 cm^–1^ are possibly attributed to the vibrations of Ag and Bg and Ag modes of Sm_2_O_3_, respectively.^29,37^ The relative intensity of the D band to the G band (*I*_D_/*I*_G_) were correlated to evaluate the degree of defect of the samples. The *I*_D_/*I*_G_ intensity ratio of the rGO/SmNPs (0.64) is higher than that observed for pure rGO (0.33), which indicates a greater number of surface defects in the rGO/SmNPs. This behavior can be attributed to the formation of a strongly bonded interaction between Sm_2_O_3_ and the rGO matrix.^29^ Based on these results, it is possible to confirm that rGO/SmNPs were successfully synthesized.

Fourier transform infrared spectroscopy (FTIR) spectra were also obtained for the rGO and rGO/SmNPs materials to evaluate the composition and potential structural modifications following the synthesis of the material. The obtained spectra are presented in Figure 4C. Analysis of the bands in both materials reveals the presence of oxygenated functional groups and the structural changes caused by the incorporation of SmNPs. In the region of 500 to 1000 cm^–1^, bands are observed that can be attributed to C–O stretching vibrations (epoxy) and possible contributions from deformed C–H bonds.^38^ In both rGO and rGO/SmNPs, these bands are visible, but in rGO/SmNPs (red), the bands are more intense, suggesting that the presence of samarium oxide is increasing the contribution of oxygenated groups and/or altering the structure of the C–O bonds.^39^ In the region of 1000 to 1500 cm^–1^, it is possible to identify vibrations associated with C–O–C bonds (ethers) and deformations of carboxylic groups. Once again, rGO/SmNPs presents more pronounced bands, which indicates a greater amount of oxygenated functional groups or interaction between samarium oxide and reduced graphene oxide.^40^ The band around 1400 cm^–1^ may be related to the angular deformation of C–OH (hydroxyls), whose intensity in rGO/SmNPs appears to be greater, suggesting an increase in the number of hydroxyl groups or an interaction with samarium. The sharp peak found at 1615 cm^–1^ is a resonance peak that can be attributed to the stretching and bending vibration of C=C and O–H groups of water molecules.^41^ In the region of 3000 to 3500 cm^–1^, it is possible to observe O–H stretching vibrations, corresponding to hydroxyl groups or water adsorbed on the surface of the material.^31,33,42^ Both spectra present bands in this region, but the intensity in rGO/SmNPs is lower, suggesting that the incorporation of samarium oxide reduces the number of hydroxyls or alters water adsorption.^43^

### 3.2 Electrochemical Investigations of rGO and rGO/SmNPs

The electrochemical performance of the rGO and rGO/SmNPs electrodes was investigated through redox behavior, change in conductivity, and electroactive area of the electrodes. For this end, measurements were carried out using cyclic voltammetry in the presence of 5.0 mmol L^–1^ [Fe­(CN)_6_]^3–/4–^ solution in 0.1 mol L^–1^ KCl at different scan rates from 10 to 200 mV s^–1^ for rGO (Figure 5A) and rGO/SmNPs (Figure 5B). A difference in the magnitude of the anodic and cathodic peak current is observed from rGO to rGO/SmNPs, which indicates that SmNPs provided a greater surface area for the redox reaction of the [Fe­(CN)_6_]^3–/4–^. This increase in current is due to some properties of samarium, such as the dielectric range from 7 to 15, and a band gap of 4.33 eV.^44^ Although the wide band gap indicates low intrinsic conductivity, the combination of SmNPs with the conductive rGO matrix forms heterojunctions that facilitate charge transfer, increase defect density, and expose new catalytic sites. This synergistic effect explains the enhanced current observed in the cyclic voltammetry of the [Fe (CN)_6_]^3–/4–^ redox system. Also, the rGO/SmNPs electrode showed lower peak separation (Δ*E* = 173 mV) when compared to the rGO (Δ*E* = 274 mV). The Ipa/Ipc ratio for rGO was 0.97 and for rGO/SmNPs was 1.03, which implies an improvement in the reversibility of the redox reaction upon modification of the electrode with SmNPs. Figure 5D shows the anodic and cathodic peak currents versus the square root of *v* for rGO and rGO/SmNPs. A linear behavior in the anodic and cathodic peak current with the scan rate indicates that the mass transport process involved for the electrodes is diffusion-controlled.^45^

**Figure 5:**
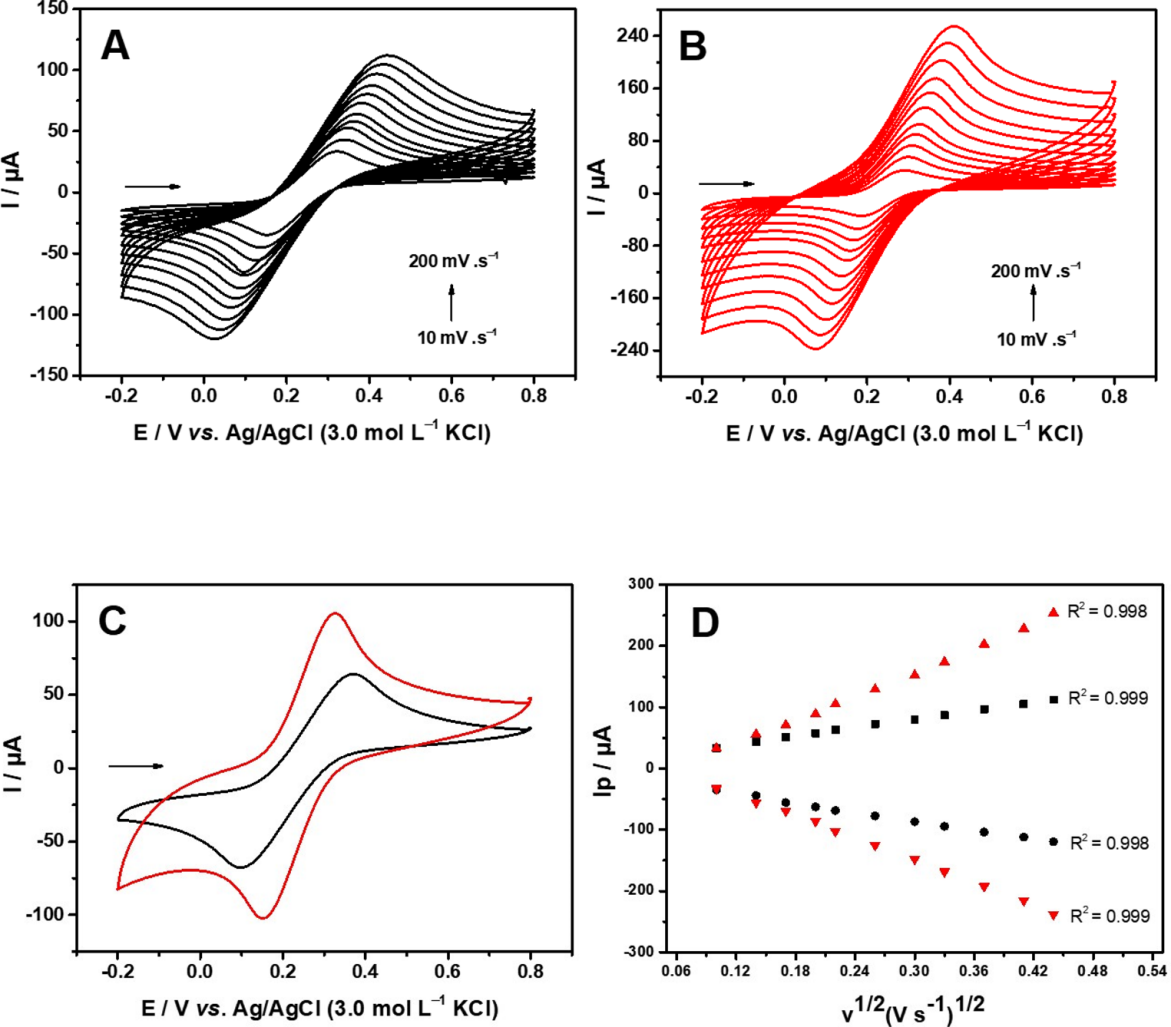
Cyclic voltammetry profiles obtained for (A) rGO and (B) rGO/SmNPs in 5.0 mmol L^–1^ [Fe­(CN)_6_]^3–/4–^ in 0.1 mol L^–1^ KCl at different scan rates (10 to 200 mV s^–1^). (C) Comparison between (black −) rGO and (red −) rGO/SmNPs in 0.1 mol L^–1^ KCl and 5.0 mmol L^–1^ [Fe­(CN)_6_]^3–/4–^ equimolar at *v* = 50 mV s^–1^. (D) Plot of *I*_p_ versus the square root of the scan rate potential (*v* ^1/2^).

The electroactive area of the rGO and rGO/SmNPs electrodes was calculated based on the Randles-Sevcik equation modified for semireversible reaction^46,47^ (eq 1), with a 5.0 mmol L^–1^ [Fe­(CN)_6_]^3–/4–^ solution in 0.1 mol L^–1^ KCl as the probe system

1 $$I_{p}=\pm 0.436n{\mathrm{FA}}_{\mathrm{ea}}C\sqrt{\frac{\mathrm{nFDv}}{\mathrm{RT}}}$$

where *I_p_* is the peak current, *F* is the Faraday constant (96485 C mol^–1^), *A*_ea_ is the electroactive area (cm^2^), *v* is the scan rate (V s^–1^), *R* is the universal gas constant (8.314 J K^–1^ mol^–1^), *T* is the temperature in Kelvin (298 K), *C* is the concentration of the redox probe (mol L^–1^), *D* is the diffusion coefficient of the redox probe (cm^2^ s^–1^) (7.6 × 10^–6^ cm^2^ s^–1^),^48^ and *n* is the number of electrons involved in the reaction. From the slope obtained in Figure 5D, the electroactive area was calculated, and values of 0.31 and 0.88 cm^2^ were obtained for the rGO and rGO/SmNPs electrodes, respectively. The presence of SmNPs led to a 2.84-fold increase in electroactive area than pure rGO. Previous work by our research team using SmNP-based materials^49^ also demonstrated a notable enlargement of the electroactive surface area, reaching 0.021 cm^2^, in contrast to 0.010 cm^2^ for the conventional graphite paste electrode. In addition, the heterogeneous electron transfer rate constant (*K* ^0^) was calculated to analyze the electron transfer behavior of the rGO and rGO/SmNPs electrodes by using the probe [Fe (CN)_6_]^3–/4–^. For this estimate, the Nicholson method^50–52^ for semireversible processes was then applied using the following eq (eq 2)­

2 $$\Psi =\pm k^{0}{\left[\pi \mathrm{DnvF}/\left(\mathrm{RT}\right)\right]}^{-1/2}$$

where (Ψ*)* is the kinetic parameter, and other constants were defined according to eq 1. As the process involves only one electron, Ψ depends on Δ*E_p_* and can be determined from the following equation (eq 3). However, if the Δ*E_p_* value exceeds 212 mV, then the *k* ^0^ constant must be calculated using the following equation (eq 4) assuming the value of α is 0.5.

3 $$\Psi =\left(0.6288+0.021\Delta E_{p}\right)/\left(1-0.0017\Delta E_{p}\right)$$

4 $$\Psi =2.18{\left(\frac{\alpha \mathrm{DnvF}}{\mathrm{RT}}\right)}^{1/2}\;\exp\left[-\left(\frac{\alpha^{2}\mathrm{nF}}{\mathrm{RT}}\right)\Delta E_{p}\right]$$

Therefore, using eqs 3 and 4, *k* ^0^ was estimated for the rGO and rGO/SmNPs electrodes by using the [Fe­(CN)_6_]^3–/4–^ redox probe. Thus, the calculated *k* ^0^ values for rGO and rGO/SmNPs were 4.14 × 10^–4^ and 4.23 × 10^–3^ cm s^–1^, respectively. The *k* ^0^ value obtained for rGO/SmNPs was approximately 10.2-fold faster than that of rGO. These observations showed that modifying the electrodes with SmNPs improves the electrocatalytic properties of the electrode, making it suitable for electrochemical analysis.

### 3.3 Analytical Response of Dopamine

The electrochemical behavior of DA was then investigated using the rGO and rGO/SmNPs electrodes through cyclic voltammetry using a 0.1 mol L^–1^ PBS buffer solution (pH 7.0). PBS adjusted to pH 7.0 was utilized, as it mimics the near-neutral conditions characteristic of human physiological systems.^53–55^ The resulting cyclic voltammograms are shown in Figure 6A. For both electrodes in a solution containing 300.0 μmol L^–1^ DA. An anodic peak, located in the 0.22 range, is associated with the oxidation process of dopamine into dopamine quinone, as represented in Figure 6B. This process involves the transfer of two electrons and two protons. During the anodic scan, the current increases proportionally to the oxidation of DA, while in the cathodic scan, a reduction peak was detected in the range of 0.16 V, corresponding to the reverse reaction of conversion of dopamine quinone to dopamine.

**Figure 6:**
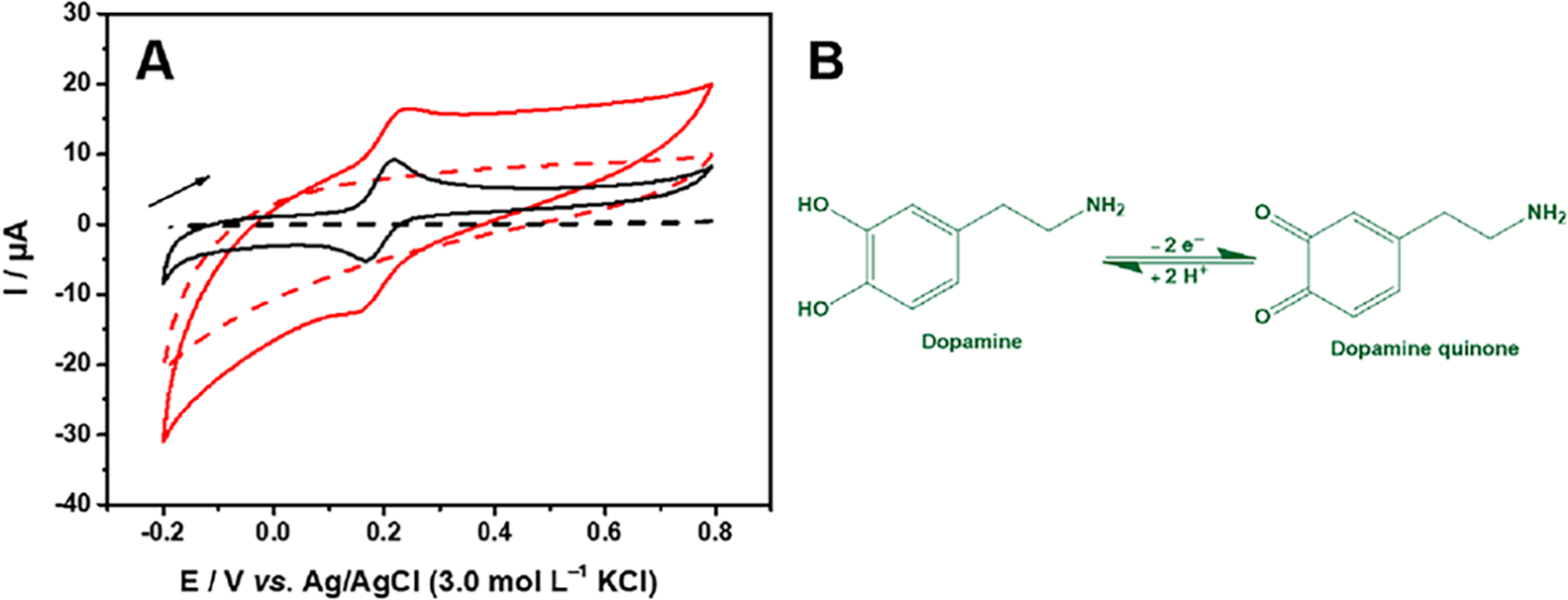
(A) Cyclic voltammograms obtained with (**−**) rGO and (**−**) rGO/SmNPs in the presence of 300.0 μmol L^–1^ and the absence of dopamine obtained in 0.1 mol L^–1^ PBS (pH 7.0) at a scan rate of 50 mV s^–1^. Dotted line: Absence of DA. (B) The electrochemical mechanism of dopamine oxidation.

However, the anodic peak current increased with the modification of Sm_2_O_3_ from 9.04 to 16.90 μA. This observation suggests that there is an increase in the interaction between DA and the high number of active sites in the rGO/SmNPs material, which could result in a highly effective electrochemical detection phase for the identification of DA. Figure 6B shows the mechanism of the electrocatalytic reaction of the DA. A marked increase in faradaic current is observed for the rGO/SmNPs electrode, demonstrating an improved electrocatalytic activity compared to rGO.

### 3.4 Analytical Performance of rGO/SmNPs

By means of this analytical technique, it was possible to verify which method was most suitable for quantifying DA using rGO/SmNPs. Consequently, the electrochemical behavior was investigated through differential pulse voltammetry (DPV) and square wave voltammetry (SWV) techniques (*n* = 3) were used in the presence of 20.0 μmol L^–1^ of DA in 0.1 mol L^–1^ PBS solution (pH 7.0). The voltammograms comparing the two techniques can be consulted in Figure S3. Pronounced peaks were observed in the same potential region around 0.2 V, which is typical for the oxidation of the DA molecule.^56^ The height and sharpness of the peaks for DPV and SWV suggested a good electrochemical response, with DA clearly detectable by both techniques. However, SWV, although with a slightly lower peak current intensity compared to DPV curves, showed a lower standard deviation for triplicate scans of 3.35%. This characteristic is crucial for reducing errors associated with false positive and false negative results in real samples, thus guaranteeing the reliability of experimental data. Therefore, an enhanced electrochemical response to DA can be observed when the SWV technique is used, which was employed for further studies. Figure S4 demonstrates the range studied for each parameter (amplitude, frequency, and step potential) and the ideal value acquired.

Under previously optimized experimental conditions for SWV, an analytical curve was constructed for increasing the concentration of DA (Figure 7A). A linear correlation between concentration, ranging from 0.5 to 20 μmol L^–1^, and peak current was observed (Figure 7B), obtaining the following equation: *I*_p_ (μA) = 1.98 ± 0.070 + (1.84 ± 0.044) *C*_dopamine_ (μmol L^–1^), with *R* ^2^ of 0.996. LOD and LOQ values were determined as 3 and 10 times the intercept standard deviation to slope ratio, respectively, based on the calibration curve.^57,58^ The values of LOD and LOQ obtained were 0.030 (±0.009) and 0.101 μmol L^–1^, respectively.

**Figure 7:**
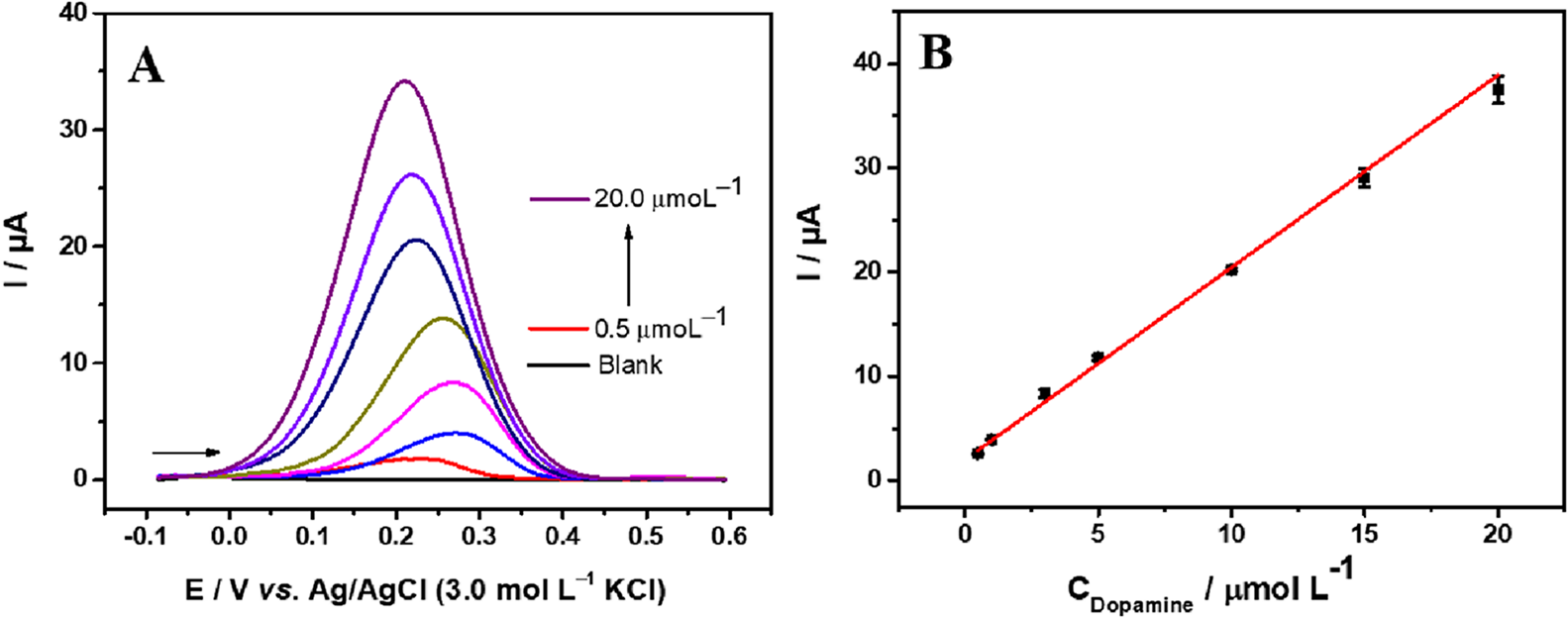
(A) Square wave voltammograms obtained in 0.1 mol L^–1^ PBS solution (pH 7.0) at varying concentrations of dopamine (0.5 to 20.0 μmol L^–1^). (B) Analytical curve for dopamine (*I*_p_ vs. *C*_dopamine_). Experimental conditions SWV: frequency = 30 Hz, amplitude = 100 mV, and step = 6 mV.

To assess the analytical efficiency of the sensor, DA detection was investigated in synthetic human serum following the addition and recovery method. Thus, three known concentrations of DA were analyzed and are shown in Table 1. Recovery values from 95.27 to 99.39% with an RSD of up to 2% indicated that there was no matrix effect due to the complexity of the composition of human serum, which contains proteins, salts, lipids, hormones, and other bioactive molecules. In complex samples, especially in biological media such as serum or plasma, biofouling, characterized by the adsorption of biomolecules on the electrode surface, represents a challenge in the field of electroanalytics, as it requires sensitivity and accuracy of the results. Therefore, Sm_2_O_3_ stands out for its resistance to passivation and biofouling,^59^ preserving the activity of the electrode surface for longer periods. Hence, it is possible to state that the presence of Sm_2_O_3_ improves the performance and precision of the new electrochemical sensors. Such results have already been reported in the literature for similar samples.^29,31^

**Table 1:** Recovery Results (*n* = 3) Obtained for Dopamine (DA) Spiked Human Serum Samples

sample	spiked (μmol L^–1^)	found (μmol L^–1^)	recovery (%)
dopamine spiked	0.5	0.49 ± 0.02	97.14 ± 1.18
	1.0	0.95 ± 0.08	95.27 ± 1.95
	3.0	2.98 ± 0.9	99.39 ± 2.03

The performance of the proposed sensor was also evaluated in repeatability and interference studies. For this, tests with 5 sensors were evaluated in the presence of 0.1 mol L^–1^ PBS at pH 7.0, including 3.0 μmol L^–1^ DA, as shown in Figure 8A. The five electrodes produced from the rGO/SmNPs material showed an RSD of 4.23%, indicating that the sensor presents an adequate reproducibility. To verify the selectivity of the rGO/SmNPs electrode, an interference test was performed for four proposed analytes commonly found in biological matrices together with DA (acetic acid (AA), uric acid (UA), glucose (GLU), and creatinine (CRE)) as shown in the presence of 0.1 mol L^–1^ PBS at pH 7.0, including 1.5 μmol L^–1^ DA, as shown in Figure 8B. For this, the tests were carried out in the presence of DA in mixture with the concomitant species. The interferent species were tested at concentrations ten times higher than that of DA concentration (1.0 μmol L^–1^). The interference level below 4% for the tested analytes demonstrates that the synthesized rGO/SmNPS presents high selectivity for the detection of DA.

**Figure 8:**
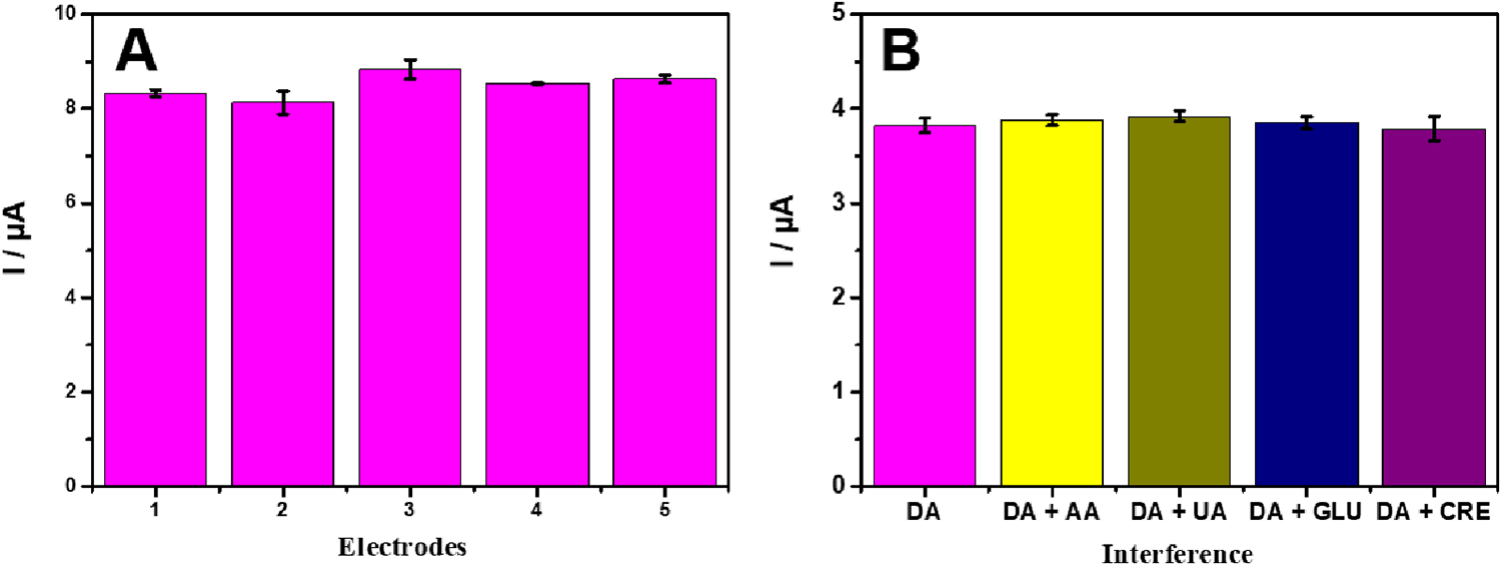
(A) Reproducibility of rGO/SmNPs electrode and (B) interference biomolecules.

The results obtained for the rGO/SmNPs electrochemical sensor in the detection of DA are compared to the other studies, as described in Table 2. In terms of preparation, the proposed sensor presented low complexity, in relation to most of the works presented that use electrospinning systems, 3D printing of electrodes, and long-term chemical syntheses. Furthermore, the sensor produced presented a detection limit compatible and/or superior to other works.

**Table 2:** Comparison of the Analytical Performance of the rGO/SmNPs Sensor for DA Detection with Previously Reported Works^a^

electrode	technique	LDR (μmol L^–1^)	LOD (μmol L^–1^)	sample application	ref
Au–Cu_2_O/rGO	DPV	10 to 90	3.9	human serum and urine	^60^
MWCNT-BPVCM-e/GCE	CV	5 to 1000	2.3	pharmaceutical	^61^
Ni@CNF/SPCE	CV	0.1 to 10	0.11	human urine and blood serum	^62^
Ti3C2/GMWCNTs/ZnO/GCE	DPV	0.01 to 30	0.0032	human serum	^63^
AuNPs@PANI core–shell	DPV	10 to 1700	5.0	human serum	^64^
PLA-G_NaOH‑30‑EC_	CV	10 to 500	3.49	urine and human serum	^65^
	DPV	7.0 to 100	2.17		
	SWV	5.0 to 100	1.67		
[PLA/PBAT]/15%GP/CPE	SWV	0.500 to 20.0	0.008	human serum	^66^
reusable graphite electrode	FIA-amperometric	2.0 to 100	0.26	synthetic urine	^67^
CuO nanowire/GCE	DPV	0.1 to 105	0.1	–	^68^
rGO_H/GCE	CV and DPV	0 to 42	0.27	–	^69^
rGO-PEDOT:PSS/GCE	CV and DPV	3 to 33	0.4	human urine	^70^
**rGO/SmNPs**	SWV	0.5 to 20	0.030	human serum	this work

a LDR: Linear dynamic range; **Au–Cu**_**2**_ **O/rGO**: Gold–Cuprous oxide/reduced graphene oxide; **MWCNT**: carbon nanotubes; **BPVCM-e:** branched amphiphilic photosensitive and electroactive polymer; **GCE:** glassy carbon electrode; **Ni@CNF**: carbon nanofiber supported nickel nanoparticles; **SPCE**: screen-printed carbon electrodes; **Ti3C2/GMWCNTs/ZnO:** sensor based on multilayer Ti3C2MXene, graphitized multiwalled carbon nanotubes and ZnO nanospheres; **AuNPs**: gold nanoparticles; **PANI**: polyaniline; **PLA-G**_**NaOH‑30‑EC**_: polylactic acid-graphene; **[PLA/PBAT]/15%GP/CPE**: conductive microfibers based on polybutylene adipate terephthalate and grafite; **CuO**: copper oxide; **PEDOT**: poly­(3,4-ethylenedioxythiophene); **PSS**: poly­(styrene-4-sulfonate).

## 4 Conclusions

An electrochemical sensing platform based on reduced graphene oxide decorated with samarium oxide nanoparticles (rGO/SmNPs) has demonstrated excellent potential for highly selective and sensitive DA determination. The obtained results demonstrated that the combination of rGO with Sm_2_O_3_ significantly improved the electrocatalytic properties of the material, increasing the electroactive area and electron transfer rate, in addition to providing high sensitivity, selectivity, and stability. These advances are essential for applications in complex biological environments, such as human serum, where the sensor showed excellent performance in terms of recovery and resistance to biomolecule interference, ensuring greater reliability for clinical analyses.

The proposed sensor also stood out in comparison with other methods described in the literature, offering a less complex preparation process and competitive performance with low detection limits and high precision. This work may contribute to the advancement of the field of electrochemical sensors, offering a promising alternative for the detection of DA in clinical and environmental applications. The use of Sm_2_O_3_ as a modifier reinforces the importance of exploring rare-earth oxides in sensors, opening the way for new studies aimed at the diagnosis and monitoring of neurotransmitters.

## Supplementary Material



## Notes

3D-printed electrochemical cell; MEV-EDS image of rGO/SmNPs; elemental mapping carbon (red), oxygen (green), gold (violet), and samarium (cyan); EDS spectra and elemental composition for rGO/SmNPs; comparison between SWV and DPV techniques in the presence of dopamine and in the absence of dopamine; optimization of the SWV technique using parameters frequency; and amplitude and step potential (PDF)
