# Supplementary material for: A New Electrochemical Sensor for Dopamine Detection Based on Reduced Graphene Oxide Modified with Samarium Oxide Nanoparticles
Source: ACS Omega. 2025 Nov 14;10(46):56290–301. doi: 10.1021/acsomega.5c08166 (PMC12658709; doi:10.1021/acsomega.5c08166)
Supplement: Supplementary file 1 [file ao5c08166_si_001.pdf]

Supporting information

A new electrochemical sensor for dopamine detection based on reduced graphene oxide modified with samarium oxide nanoparticles

Rodrigo Vieira Blasques[1], Vinicius Aparecido Pedro Oliani da Silva[2], Amanda Caroline Nascimento Sousa[1], Tatiana Maria Barreto de Freitas[1], Leliz Ticona Arenas[3], Glauber Cruz[4], Marcelo Barcellos da Rosa[5], Gabriel Braga Marques Teobaldo[7], Matheus Henrique Martins[6], Fabio Luiz Pisseti[6], Rita de Cássia Mendonça de Miranda[1], Luís Cláudio Nascimento da Silva[1], Paulo César Mendes Villis[1]*

[1]*Electrochemistry and Biotechnology Laboratory, University of CEUMA - UNICEUMA, 65065-470, São Luís, MA, Brazil*

[2]*Laboratory of Sensors, Nanomedicine and Nanostructured Materials, Federal University of São Carlos, Araras, 13600-970, Brazil*

[3]*Laboratory of Solids and Surfaces, Institute of Chemistry, Federal University of Rio Grande do Sul – UFRGS, 91501-970, Porto Alegre, RS, Brazil*

[4]*Processes and Thermochemical Systems Laboratory (LPSisTer), Department of Mechanical Engineering, Federal University of Maranhão (UFMA), São Luís, Maranhão, Brazil*

[5]*Federal University of Santa Maria - UFSM, Department of Chemistry, Av. Roraima, 1000, 97105-900, Santa Maria, RS, Brazil*

[6]*Institute of Chemistry, Federal University of Alfenas, Rua Gabriel Monteiro da Silva, 700, Alfenas-MG, 37130-001, Brazil.*

[7]*Institute of Physics, University of São Paulo, CEP: 05508-090*

*Corresponding authors: blasques@live.com (R. V. Blasques)

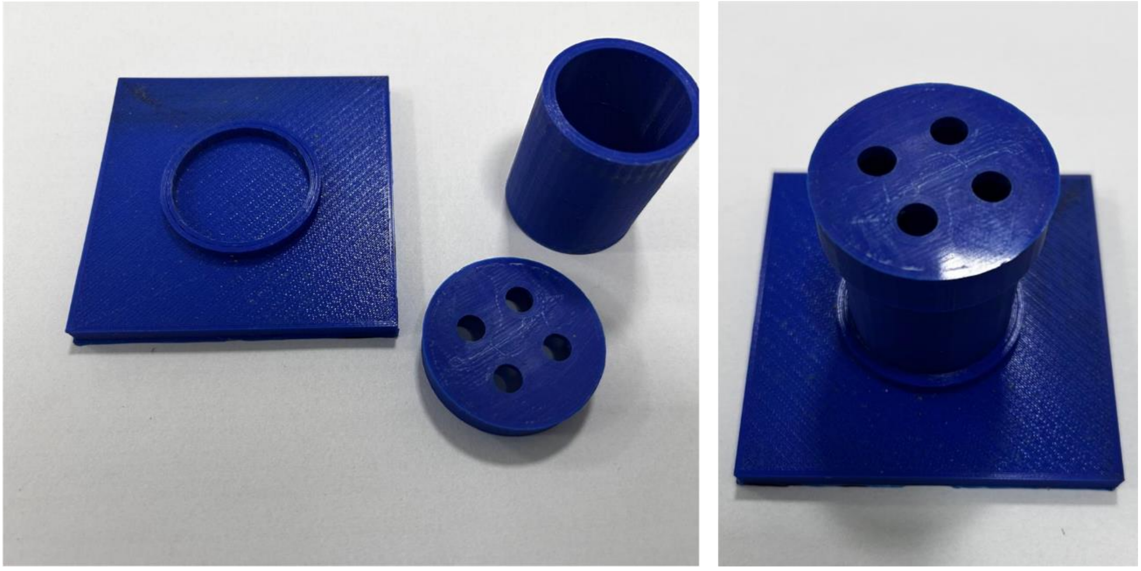

Fig. S1. 3D printed electrochemical cell.

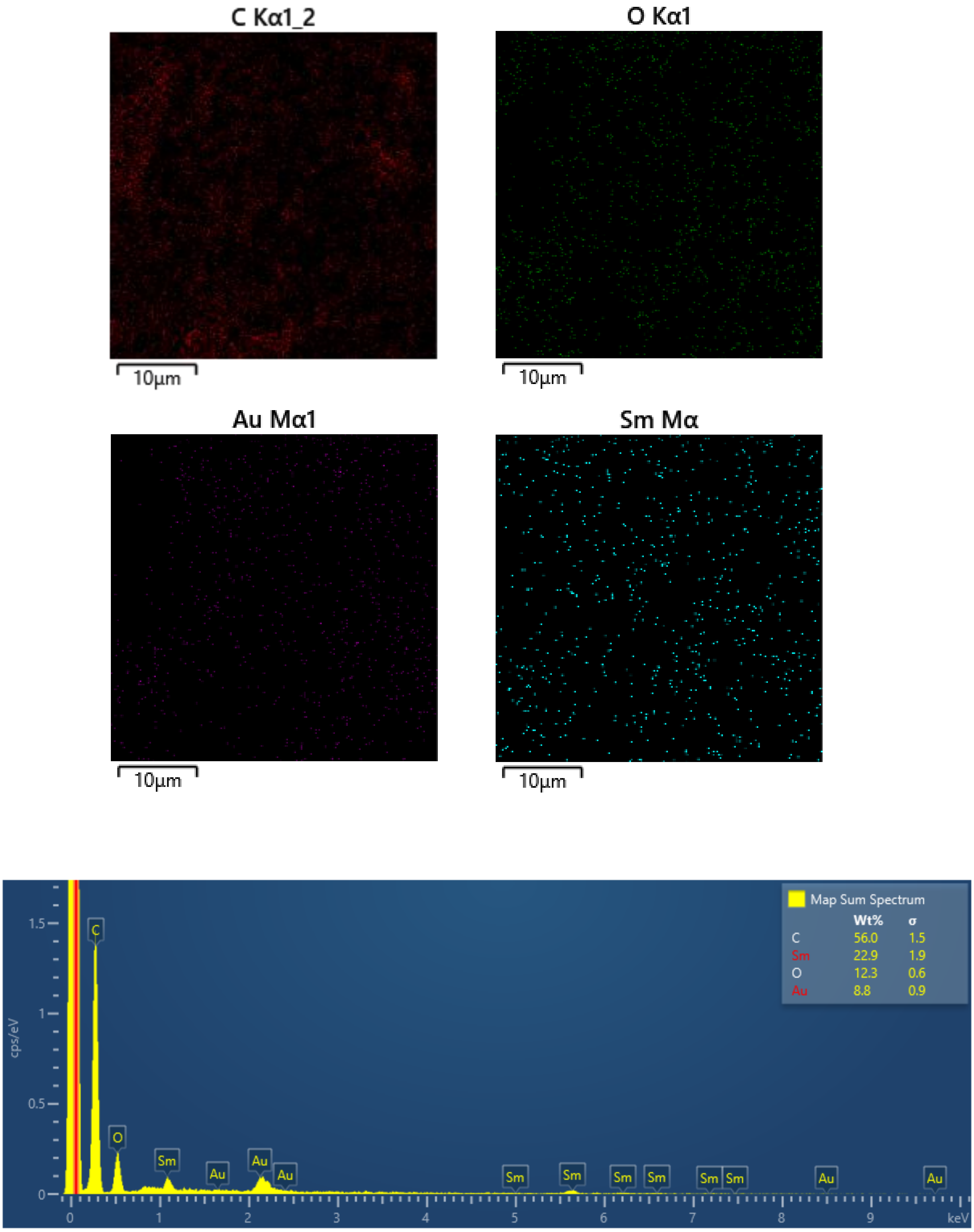

Fig. S2. MEV-EDS image of rGO/SmNPs. Elemental mapping carbon (red), oxygen (green), gold (violet) and samarium (cyan). EDS spectra and elemental composition for rGO/SmNPs.

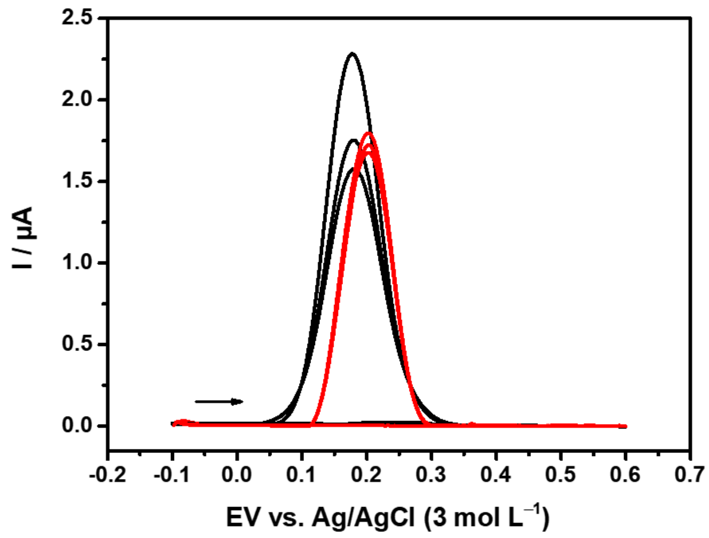

Fig. S3. Comparison between SWV (–) and DPV (–) techniques in the presence of 20.0 µmol L^{-1} Dopamine and in the absence of Dopamine (black and red lines), using a 0.1 mol L^{-1} PBS solution (pH 7.0) and the proposed electrode. SWV parameters: f = 10 Hz, a = 50 mV, step 1 mV; DPV parameters: a = 50 mV, modulation time = 25 ms, v = 10 mV s^{-1}.

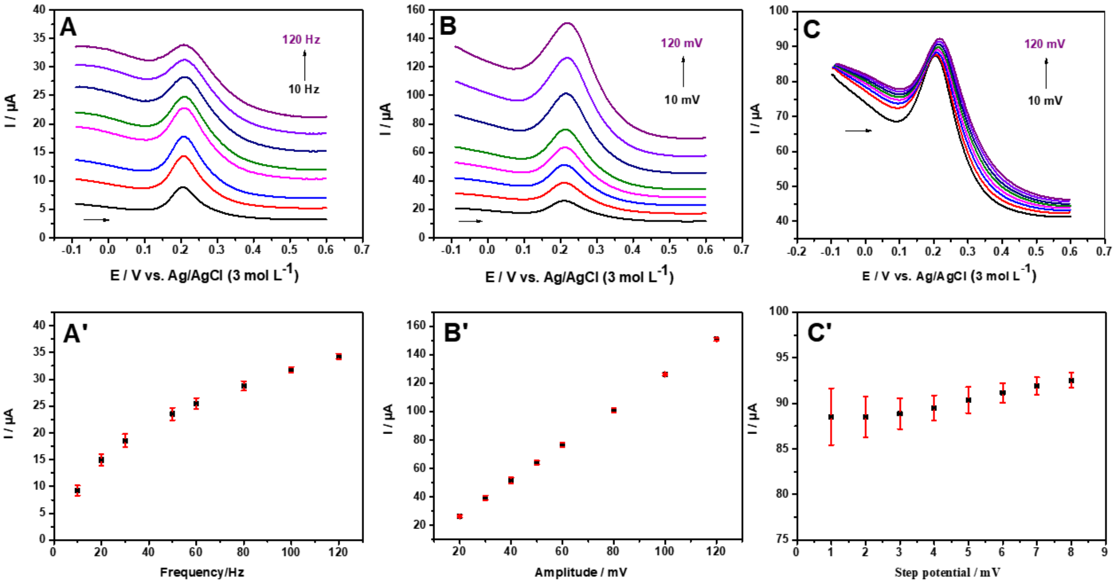

Fig. S4. SWV response for 20.0 µmol L^{-1} Dopamine in 0.1 mol L^{-1} PBS solution (pH 7.0) obtained at different (A) frequency (from 10 to 120 mV), (B) amplitude (from 20 to 120 mV) and (C) step potential (from 1 to 8 mV), and respective plots of peak current *versus* varied parameters (A'-C').
